# Central serous chorioretinopathy and endogenous cortisol – is there an association?

**DOI:** 10.4103/0301-4738.67055

**Published:** 2010

**Authors:** Charudatt Chalisgaonkar, Siddharth Chouhan, Sujata Lakhtakia, Pankaj Choudhary, P C Dwivedi, M K Rathore

**Affiliations:** Department of Ophthalmology, GM Hospital & SS Medical College, Rewa (MP), India

Dear Editor,

We read the article of Zakir *et al*.[1] titled ″Serum cortisol and testosterone levels in idiopathic central serous chorioretinopathy″ with interest, published in the *Indian Journal of Ophthalmology*.

In their study, the mean serum cortisol value of 23 central serous chorioretinopathy (CSCR) patients was compared with that of only 12 controls. For the best comparison of mean values, the number of test and control subjects should be same. In their study only two out of 23 CSCR patients were having serum cortisol levels outside normal limit. Though the mean serum cortisol value of CSCR patients was found to be higher than that of control patients, endogenous cortisol may not act pathologically unless it is outside normal range. Further, only morning blood samples were investigated. This may affect the final mean cortisol values of study subjects as endogenous cortisol shows diurnal variations. This fluctuation varies from individual to individual. Hence for accurate assessment of endogenous cortisol, both morning and evening samples should be taken.

We also have conducted a similar study but with opposite result titled ″Central serous chorioretinopathy and endogenous cortisol – is there an association?″ which was presented as a free paper at the Madhya Pradesh State Ophthalmic Society (MPSOS) conference, October 2009, held at Bhopal.

In our study we compared serum cortisol levels of 37 consecutive patients of acute CSCR and 32 patients presenting with sudden painless diminution of vision without CSCR as controls. Patients with conditions altering the endogenous cortisol levels were excluded from the study.

In our study serum cortisol estimation was done by Chemi-luminescent Immuno-assay taking venous blood samples at 8.00 am and 4.00 pm from both groups. The mean value of 8.00 a.m. serum cortisol in test group was 8.76 µg/dl compared to 8.53 µg/dl in control group, the difference being statistically not significant (*P*=0.975). The mean value of 4.00 p.m. serum cortisol in CSCR patients was 6.13 µg/dl and 6.07 µg/dl in controls. This difference was also statistically insignificant (*P*=0.948). Our observations are in accordance with those of Haimovici *et al*.[2] and Akira *et al*.[3] The corticosteroids generally strengthen tight junctions and reduce blood-brain and blood-retinal barrier breakdown. They also decrease vascular permeability. As such they should have no role to play in the pathogenesis of CSCR. But authors supporting the positive role of cortisol in CSCR postulate that cortisol paradoxically may cause increased angiographically documented retinal pigment epithelium and choroidal permeability.[4] However, the precise mechanism regarding their direct contribution to the development or worsening of CSCR remains speculative.

Our study did not find a precise correlation of serum cortisol with CSCR. However, continued attention to this clinically interesting disease entity in terms of endocrinological association may lead to its better understanding in the future. Till such time, it can be said that the association of endogenous cortisol with CSCR, though probable still remains inconsistent and there may be other contributory pathogenic mechanisms which need further evaluation.
